# Seasonal screening of pesticide residues in beehive products collected from different districts in Egypt

**DOI:** 10.1007/s10661-024-12451-2

**Published:** 2024-02-22

**Authors:** Atef M. K. Nassar, Yehia M. Salim, Eman Nour-Eldeen, Mohamed S. Younis, Mahmoud M. Kelany, Mohamed A. Shebl, Abdallah S. Shafey, Hossam F. Abou-Shaara

**Affiliations:** 1 Department of Plant Protection, Faculty of Agriculture, Damanhour University, P.O. Box 22516, Damanhour, Egypt; 2 Bee Research Department, Plant Protection Research Institute, Agricultural Research Center, Cairo, Egypt; 3 Plant Protection Department, Desert Research Center, Elamriya, Alexandria, Egypt; 4 Plant Protection Department, Faculty of Agriculture, Suez Canal University, Ismailia, 41522 Egypt

**Keywords:** Pesticides residues, Honey, Beeswax, Pollen, Chemical contaminants

## Abstract

Pesticides are of immense importance in agriculture, but they might contaminate bees’ products. In this study, samples of honey, pollen, and beeswax were collected, seasonally, from apiaries in Toshka (Aswan), El-Noubariya (El-Beheira), and Ismailia (Ismailia) cities in Egypt. The pesticide residues were analyzed using the GC-MS after being extracted and cleaned using the QuEChERS method. Results showed that samples from El-Noubariya had great content of residues followed by Ismailia, and finally Toshka. Samples collected during fall and winter had the highest pesticide residue contents. Specifically, the phenylconazole fungicide group was repeatedly detected in all the examined samples along with organophosphate insecticides. Beeswax samples had the greatest amounts of pesticide residues followed by pollen and then honey samples. Chlorpyrifos (0.07–39.16 ng/g) and profenofos (1.94–17.00 ng/g) were detected in honey samples and their products. Pyriproxyfen (57.12 ng/g) and chlorpyrifos-methyl (39.16 ng/g) were detected in great amounts in beeswax samples from Ismailia and El-Noubariya, respectively. Yet, according to health hazard and quotient studies, the amounts of pesticides detected in honey do not pose any health threats to humans.

## Introduction

Honeybees play a critical role as plant pollinators and in honey production. Bees pollinate about 34% of plants that contribute significantly to the total human dietary supply (Breeze et al., 2011) and provide an added value to crop productivity and quality (Khalifa et al., 2021). They secure about 9.5% of the total economic revenue of farm products, which accounts for 200 billion USD globally, where they pollinate more than 100 cash crops (Hristov et al., 2020). In Egypt, there are about 1.3 million hives and 270,000 beekeepers (Abou-Shaara, 2009).

Every day, worker bees fly about 10 flies to harvest flowers’ syrup, water, and pollen from an area of about 7 km^2^ surrounding the hive (Fernandez et al., 2002). After their daily activity, honey bees combine the nectar and secretions and deposit them for dehydration in honeycombs for ripeness and maturation of honey (Shafiee et al., 2013). Based on floral sources, honey is comprised of carbohydrates, health-related secondary chemicals, minerals, proteins, sugars, and vitamins (Santos-Buelga & González-Paramás, 2017). Therefore, it has many therapeutic effects including antibacterial, antiparasitic, antimutagenic, and antitumor. Also, it was reported to reduce cardiovascular diseases since early times when it was consumed in old Egyptian, Greek, Indian, and Roman civilizations (El-Nahhal, 2020).

It is generally accepted that honey and beehive products are clean and healthy. However, nowadays, it is polluted with different contaminants either from the environment or the hive (Al-Waili et al., 2012; Andreo-Martínez et al., 2020; Bogdanov, 2006; Chauzat & Faucon, 2007; Rodríguez López et al., 2014). During the process of foraging, bee workers captured diverse chemical pollutants and suspended materials from the air (Fernandez et al., 2002). Honey contaminants might include heavy metals, polychlorinated biphenyls, antibiotics, organic acids, polyaromatic compounds originating from oil, and pesticides (Bogdanov, 2006; Davodpour et al., 2019).

Also, the application of these chemicals in the nearby fields might impose a risk of contaminating honey and other products (Bogdanov, 2006; Johnson et al., 2010; Rodríguez López et al., 2014; Tsipi & Hiskia, 1999; Wallner, 1999). Additionally, indirect exposure to widespread and extensively used pesticides might accumulate residues in bees’ products during blossom seeking (Panseri et al., 2014). Therefore, the detection of pesticide residues in bees’ products is routinely accomplished and is gaining increased attention in recent years. For example, residues of organochlorine and synthetic pyrethroids were reported in honey samples. Hexachlorobenzene, permethrin, and heptachlor epoxide residues were the major detected amounts (Malhat et al., 2015). Tau-fluvalinate pesticide residue was detected only in one sample out of 64 honey samples from different cities in Egypt (Shendy et al., 2016).

Moreover, the detection of various contaminants in hive products differs in quantity and type, for example, beeswax samples showed high concentration of antibiotics, persistent lipophilic acaricides, and lead compared other products (honey, pollen, propolis, and royal jelly) (Bogdanov, 2006). Acaricides, fungicides, insecticides, and herbicides were reported in honey and its products (Bogdanov, 2006; Calatayud-Vernich et al., 2018; de Pinheiro et al., 2020; Irungu et al., 2016; Panseri et al., 2014; Pohorecka et al., 2012; Wang et al., 2022). Various groups of insecticides including organochlorine, organophosphates, carbamates, pyrethroids, phenylpyrazoles, and neonicotinoids are used inside the apiary to manage disease agents (Al-Naggar et al., 2015).

Contamination of honey with pesticide residues is a worldwide problem. For example, Peruvian honey samples had residues of organohalogen and organophosphorus insecticides (Rissato et al., 2007). Additionally, testing hive produces for the presence of pesticide residues revealed the detection of 60, 30, and 17 insecticides, fungicides, and herbicides in 887 US samples of beeswax and pollen (Johnson et al., 2010). Rodríguez López et al. indicated that honey samples from Colombian markets had residues of γ-HCH, HCB, chlorpyrifos (CPF), and fenitrothion (Rodríguez López et al., 2014). In another study, about 17 pesticides residues were detected in commercial honey samples collected from Kenya and Ethiopia at low levels except for malathion which was detected at high concentrations (Irungu et al., 2016). Coumaphos, CPF, dimethoate, and imidacloprid pesticides were the most frequently detected in honey samples from Spain (Calatayud-Vernich et al., 2016). Twenty-five out of 35 honey samples collected from Brazilian markets had residues of monocrotophos, trichlorfon, and chlorpyrifos-methyl (de Pinheiro et al., 2020).

Interestingly, the location of beehives might affect the levels of pesticide residues in its products. For example, honey samples from apiaries located in industrial areas had high amounts of DDT, DDD, and DDE residues, while samples collected from beehives near apple orchards had CPF and quinoxyfen residues, and samples from mountain hives were clean (Panseri et al., 2014). Moreover, bees’ goods differed significantly in their content of pesticide residues. Pollen samples contained greater concentrations of profenofos, CPF, malathion, and diazinon insecticides compared to honey samples (Al-Naggar et al., 2015). After the exposures to pesticides, beeswax had greater amounts of residues compared to other products (Johnson et al., 2010). Also, beeswax showed residues of pesticides belonging to organophosphates, pyrethroids, organochlorine, and antibiotics were found in samples collected from France (Chauzat & Faucon, 2007) and Spain (Calatayud-Vernich et al., 2018). Bee bread samples were contaminated with glyphosate, mandipropamid, tau-fluvalinate, metalaxil, and spiroxamine pesticides (Bergero et al., 2021). Also, miticides and insecticides (mainly, CPF and acetamiprid) were detected in pollens (Calatayud-Vernich et al., 2018). Also, lambda-cyhalothrin and bifenthrin insecticides were found in beebread samples (Lee & Lee, 2015). Contamination of beehive products with residues might originate from plants treated with pesticides. After the application of thiamethoxam, thiacloprid, and acetamiprid as a seed treatment on rape, their residues were greater in the nectar compared to pollen samples (Pohorecka et al., 2012).

Honey is widely considered and accepted as a clean and healthy food, but its contamination might pose health hazards to consumers and the survival of pollinators (Panseri et al., 2014; Wang et al., 2022). Hence, it is vital to study the potential health impacts of pesticide residues to consumers through hazard assessments. Measurements of health risks consider potency of exposure and dietary intake. The hazard quotient (HQ) method was employed to assess the human risk of pesticide residues in honey (Al-Naggar et al., 2015; Eissa et al., 2014; El-Nahhal, 2020; Végh et al., 2023). Where, these residues might affect consumers’ health; therefore, monitoring them in honey and hive products is required to introduce safe food for human (Eissa et al., 2014; Fernandez et al., 2002). Thus, this research project was conducted to evaluate the residues of pesticides in honey, pollen, and beeswax during the four seasons of the year from bee colonies placed in three locations in Egypt: El-Noubariya, Ismailia (North), and Toshka (South).

## Materials and methods

### Sampling location

Honey, pollen, and beeswax samples were collected from natural honeycombs from beehives located in El-Noubariya (El-Beheira Governorate, North), Ismailia (Ismailia Governorate, North-East), and Toshka (Aswan Governorate, South) during the spring, summer, and autumn seasons of 2021 and winter season of 2022. Exactly, 5 blocks (20 × 20 cm) of honeycombs, at each collection time, were transferred to the laboratory in clean Ziplock pages and used to collect honey, pollen, and beeswax with few hours of collection.

### Standard chemicals

Certified reference materials of pesticides (Table 1; pesticides were selected based on the results of pre-study field survey) were used for quality control parameters (recovery percentages, limits of detection (LOD) and quantification (LOQ), coefficients of variability percentages (CV%) expressed as inter- and intra-assay), standard curves, and spiking of samples with no detected residues. Kits of QuEChERS of solid phase extraction (SPE) and clean-up and HPLC-grade acetonitrile and acetic acid were bought from Agilent Technologies through local providers.

**Table 1:** List of detected pesticides, their limits of detection (LOD; ng/g), limits of quantification (LOQ; ng/g), and coefficients of variability (CV%) expressed as inter- and intra-assay values using GC-MS

Rt (min)	Common name	*Use	LOD (ng/g)	LOQ (ng/g)	CV%	
					Intra-assay	Inter-assay
6.854	Azinphos-ethyl	I	1.11	3.35	4.34	11.62
10.907	Pyrimethanil	F	1.39	4.17	3.58	9.61
10.918	Atrazine	H	0.56	1.77	4.53	7.82
11.489	Diazinon	A, I	1.11	3.36	4.19	8.17
11.583	Malathion	A, I	0.83	2.50	3.89	5.04
12.935	Metribuzin	H	1.39	4.18	2.92	4.15
13.481	Profenofos	A, I	0.83	2.50	4.36	6.15
13.689	Metalaxyl	F	1.11	3.45	5.32	10.35
14.681	Triazophos	A, I, N	0.56	1.16	3.49	7.59
15.302	Chlorpyrifos-methyl	I	0.83	2.50	4.08	8.27
15.635	Imazalil	F	1.67	5.00	3.37	6.27
15.909	Iprodione	F	1.11	3.34	3.18	8.31
16.799	Pendimethalin	H	1.67	5.00	3.75	8.11
16.894	Penconazole	F	1.67	5.00	3.51	5.19
16.902	γ-Cyhalothrin	I	0.28	0.83	3.62	9.85
18.712	Cypermethrin	I	1.39	4.16	3.98	6.55
20.272	Myclobutanil	F	0.83	2.50	3.04	4.32
20.307	Flusilazole	F	1.11	3.37	3.86	6.45
20.454	Fludioxonil	F	1.39	4.17	3.76	5.87
20.562	Fenvalerate	I	0.56	1.68	5.14	7.54
23.370	Propiconazole	F	0.83	2.50	5.77	4.24
24.418	Propagite	A	1.39	4.17	3.68	7.48
27.873	Pyriproxyfen	I	0.83	2.50	3.34	5.63

Limits of detection (LODs) of pesticides were defined based on a signal three times the background noise (S/N×3), and limits of quantification (LOQs) of pesticides were calculated based on a signal ten times the background noise (S/N×10)

*A acaricide, *F* fungicide, *H* herbicide, *I* insecticide, *N* nematicide

### Quality traits and recovery studies of GC-MS responses

Recovery percentages were calculated by spiking organic honey, pollen, and beeswax samples with pesticide standard samples at levels equal to LOQ and 2X LOQ in ng/g; each concentration was repeated three times. Accuracy of the employed analytical method was calculated by the analysis of spiked honey, pollen, and beeswax samples. The quality assurance parameters were validated and performed following the directions of the European Commission regulation document no. SANTE/11312/2021 (European Commission, 2021; World Health Organization, 2014).

### Estimation of pesticide residues

#### Extraction and clean-up

Extraction of pesticide residues was completed using a modified QuEChERS method (Anastassiades et al., 2003; Eissa et al., 2014). Five grams of honey, pollen, and beeswax samples were extracted with 10 mL of acetonitrile (0.1% glacial acetic acid) and vigorously shaken for 10 min by the vortex mixer. Then, 4 g of magnesium sulfate (anhy.), sodium chloride (1 g), and 50 μL of the internal standard (TPP) were mixed with samples extracts for 1 min and centrifuged under cooling for 10 min at 1268 × g (Hermle Labortechnik GmbH, Siemensstr25 D-78564 Wehingen, Germany)**.** Then, 1 mL of the acetonitrile layer was cleaned up with PSA (25 mg) and MgSO_4_ (150 mg) and centrifuged for 5 min at 1268 × g. Supernatants (about 0.5 mL) of each extract were injected into GC-MS for analysis**.** The clean-up process of honey samples was completed using double the amounts of PSA and MgSO_4_ to reduce interferences.

#### GC-MS analysis

Extracted honey, pollen, and beeswax samples were injected into the 7890B Agilent GC system connected to 5977A MS instrument. The system was operated by Chem Station software, and data acquisition was completed with Mass Hunter software. Separation was done using an HP-5MS capillary column (30 m × 0.53 mm i.d. 0.25 μm film thickness) and helium the carrier gas (1.1 mL/min flow rate). Equipment programming was achieved based on the AOAC, 2007 as the following: the column temperature was initially set at 80 °C for 6 min, raised to 215 °C at 15 °C/min (hold for 1 min), then to 230 °C at 5 °C/min, and to 290 °C at 5 °C/min (hold for 2 min). The pesticides were identified by both the full mass spectrum scans and retention time (total ion chromatogram (TIC)) in comparison with the standard concentrations of each pesticide.

### Estimation of potential health risk of pesticide residues

Mean daily intake (MDI) value of each pesticide was estimated using the following equation: MDI = (PS × Q)/BW, where PS is the average amount of each residue in ng/g, *Q* is the daily consumed amount of honey by an adult, which is 0.8 g/kg body weight/day (Australian Pesticides and Veterinary Medicines Authority, 2023), and BW is the body weight of an adult consumer (60 kg) (EFSA, 2007). The health quotient (HQ) was estimated using HQ = MDI/ARfD formula, where ARfD is acute reference dose of each pesticide residue in ng/g/day (Australian Pesticides and Veterinary Medicines Authority, 2023; El-Nahhal, 2020; Choudhury & United States Environment Protection Agency, 2000; WHO, 2012).

### Statistical analysis

Pesticide residue results were statistically analyzed using PROC GLM of the Statistical Analysis System software version 9.3 (SAS, Cary, USA) and presented as mean ± SD.

## Results

### Detection limits, quality traits, and recovery examinations

The GC-MS detection limits (LOD) were from 1.65 to 9.90 ng/g and limits of quantifications (LOQ) ranged from 5 to 30 ng/g for the examined pesticides (Table 1). The employed analytical method was linear within a range of 10–500 ng/g depending on the tested pesticide with correlation coefficients of more than 0.996. Recovery studies of examined pesticides were completed at two levels (LOQ and 2X LOQ). The CV% expressed as intra- (from 2.92 to 5.77%) and inter-assay (from 4.15 to 11.62%) were less than (21%) of the reported CV% by (Shendy et al., 2016) and the limits defined by ICH for the suitability of the analytical method (Branch, 2005).

**Fig. 1:**
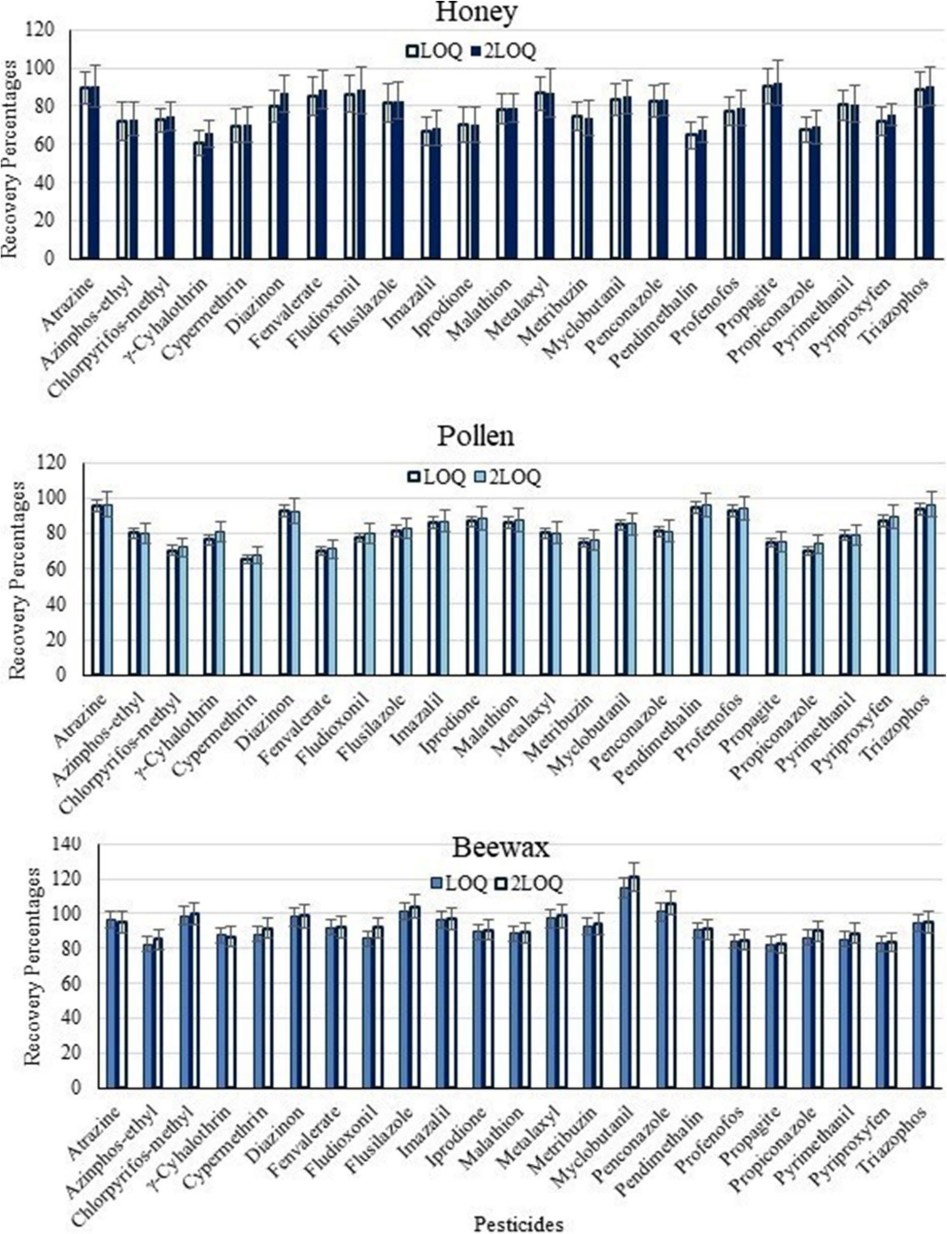
Recovery percentages of detected pesticides using GC-MS in honey, pollen, and beeswax samples collected from El-Noubariya, Ismailia, and Toshka cities during the spring, summer, and autumn of 2021 and winter of 2022. Two concentrations were studies equal to LOQ (low concentration) and 2X LOQ (high concentration) and error bars equal to the relative standard deviation (RSD%)

Results showed that at the LOQ level, recovery % ranged from 60.74% for λ-cyhalothrin to 90.45% for propargite from honey samples (Table 1). For pollen samples, recoveries ranged from 65.54% (cypermethrin) to 95.12% (atrazine). For beeswax samples, recoveries ranged from 82.00 to 114.55% for azinphos-ethyl and myclobutanil, respectively. At the 2X LOQ level, recovery percentages of pesticides ranged from 65.43 to 91.93%, 68.02 to 96.21%, and 85.20 to 120.97% from honey, pollen, and beeswax samples, respectively (Fig. 1). These results were similar to other results by several researchers who reported recoveries from 68 to 126% (Blasco et al., 2004).

### Pesticides residue levels

#### Spring season

During spring 2021, about five pesticides were found in the bee products collected from El-Noubariya, pollen and beeswax from Ismailia, and no pesticides were detected in all products collected from Toshka (Table 2). Pollen samples from El-Noubariya had 0.07 ± 0.001, 0.54 ± 0.002, and 3.79 ± 0.017 ng/g of chlorpyrifos, fludioxonil, and profenofos, respectively. Beeswax had residues of chlorpyrifos and fludioxonil at 1.14 ± 0.002 and 1.73 ± 0.001 ng/g, respectively, while only flusilazole residues (0.61 ± 0.031 ng/g) were detected in honey. Residues of chlorpyrifos, fludioxonil, and flusilazole were detected in pollen samples from Ismailia at 1.73 ± 0.001, 0.85 ± 0.022, and 1.24 ± 0.003 ng/g, respectively. Only penconazole residues were found in beeswax samples (6.27 ± 0.031 ng/g) from Ismailia, and no pesticides were discovered in honey samples from Ismailia. All three beehive products from Toshka were clean of pesticides at the limits quantification of the employed GC-MS.

**Table 2:** Mean ± SD of detected pesticides (ng/g) in samples collected from bee colonies in the three studied locations during the spring season of 2021

RT (min)	Pesticide	El-Noubariya			Ismailia			Toshka		
		Pollen	Beeswax	Honey	Pollen	Beeswax	Honey	Pollen	Beeswax	Honey
13.481	Profenofos	3.79 ± 0.017	-	-	-	-	-	-	-	-
15.302	Chlorpyrifos-methyl	0.07 ± 0.001	1.14 ± 0.002	-	1.73 ± 0.001	-	-	-	-	-
16.894	Penconazole	-	-	-	-	6.27±0.031	-	-	-	-
20.307	Flusilazole	-	-	0.61 ± 0.002	1.24 ± 0.003	-	-	-	-	-
20.454	Fludioxonil	0.54 ± 0.002	1.73 ± 0.001	-	0.85 ± 0.022	-	-	-	-	-
Total		4.40	2.87	0.61	3.82	6.27	-	-	-	-

Data presented as mean ± standard deviation

-Not detected

#### Summer season

About eight pesticides were reported in samples gathered from the three examined locations except for honey samples from Toshka, where no pesticides were detected (Table 3). Malathion, chlorpyrifos-methyl, fludioxonil, flusilazole, myclobutanil, penconazole, profenofos, and propiconazole were identified in pollen, beeswax, and honey samples from El-Noubariya. Levels of pesticides in pollen, beeswax, and honey samples were from 1.03 ± 0.001 to 6.71 ± 0.032, 7.45 ± 0.211 to 18.70 ± 1.041, and 0.21 ± 0.001 to 2.11 ± 0.011 ng/g, respectively. Six out of eight pesticides were detected in samples collected from Ismailia including chlorpyrifos-methyl, fludioxonil, flusilazole, profenofos, propiconazole, and penconazole. Amounts ranged from 1.73 ± 0.001 to 4.51 ± 0.020 ng/g in pollen, from 1.94 ± 0.004 to 5.87 ± 0.041 ng/g in beeswax, and from 0.21 ± 0.001 to 0.81 ± 0.001 ng/g in honey samples. Less number and concentrations of pesticides were found in pollen and beeswax collected from Toshka. Levels of residues in pollen ranged from 0.09 ± 0.001 (chlorpyrifos-methyl) to 0.98 ± 0.001 (propiconazole) ng/g while the detected amounts in beeswax ranged from 0.67 ± 0.001 (penconazole) to 2.07 ± 0.002 (propiconazole) ng/g.

**Table 3:** Mean ± SD of detected pesticides (ng/g) in samples collected from bee colonies in the studied locations in the summer season of 2021

RT (min)	Pesticide	El-Noubariya			Ismailia			Toshka		
		Pollen	Beeswax	Honey	Pollen	Beeswax	Honey	Pollen	Beeswax	Honey
11.583	Malathion	-	8.73 ± 0.048	-	-	-	-	-	1.11 ± 0.002	-
13.481	Profenofos	-	10.14 ± 0.42	-	-	1.94 ± 0.004	-	-	-	-
15.302	Chlorpyrifos-methyl	1.03 ± 0.001	-	2.11 ± 0.011	1.73 ± 0.001	-	0.81 ± 0.001	-	-	-
16.894	Penconazole	3.21 ± 0.002	-	-	2.31 ± 0.003	-	-	0.09 ± 0.001	0.67 ± 0.001	-
20.272	Myclobutanil	6.71 ± 0.032	-	-	-	-	-	-	-	-
20.307	Flusilazole	-	18.70 ± 1.041	2.01 ± 0.001	-	5.87 ± 0.041	-	-	1.27 ± 0.002	-
20.454	Fludioxonil	5.41 ± 0.022	-	0.21 ± 0.001	4.51 ± 0.02	-	0.21 ± 0.001	-	-	-
23.370	Propiconazole	-	7.45 ± 0.211	-	-	4.37 ± 0.031	-	0.98 ± 0.001	2.07 ± 0.002	-
Total		16.36	45.02	4.33	8.55	12.18	1.02	1.07	5.12	-

Data presented as mean ± standard deviation

-Not detected

#### Autumn season

Data in Table 4 showed the residues that were reported in the analyzed samples gathered from the three locations during the fall season of 2021. Ismailia had a high number of pesticides (12), followed by El-Noubariya (11), and finally Toshka (6). The detected pesticides in pollen samples were azinphos-ethyl, diazinon, propargite, profenofos, metalaxyl, triazophos, chlorpyrifos-methyl, pendimethalin, cypermethrin, metribuzin, and penconazole ranging from 1.14 ± 0.011 to 37.22 ± 1.600 ng/g. The same pesticides were detected in beeswax in addition to flusilazole and pyriproxyfen, ranging from 0.80 ± 0.122 to 39.16 ± 1.013 ng/g. Pesticide residues in honey ranged from 3.88 ± 1.068 (azinphos-ethyl) to 34.18 ± 10.156 (chlorpyrifos-methyl) ng/g. Greater amounts of pesticides were found in beeswax samples from Ismailia, where it ranged from 1.57 ± 0.567 (profenofos) to 57.12 ± 11.246 (pyriproxyfen) ng/g. About 1.185 ± 0.199 (cypermethrin) to 30.05 ± 0.920 ng/g (chlorpyrifos-methyl) were detected in pollen sample. For the honey samples, flusilazole was the least detected (1.21 ± 0.238 ng/g), and chlorpyrifos was the greatest detected (21.26 ± 3.665 ng/g) pesticides. Residues of propargite and chlorpyrifos-methyl were found in pollen and beeswax samples; pendimethalin was detected in pollen; penconazole was found in beeswax samples, and metalaxyl and pyriproxyfen were in honey samples. In the three tested products, the amounts of pesticides ranged from 0.89 ± 0.263 to 15.80 ± 2.934 ng/g.

**Table 4:** Pesticide residues (ng/g) in pollen, beeswax, and honey samples collected from studied locations during the fall season of 2021

Rt (min)	Pesticide	El-Noubariya			Ismailia			Toshka		
		Pollen	Beeswax	Honey	Pollen	Beeswax	Honey	Pollen	Beeswax	Honey
6.854	Azinphos-ethyl	14.42 ± 2.599	13.41 ± 2.752	3.88 ± 1.068	-	26.76 ± 4.351	-	-	-	-
10.907	Pyrimethanil	-	-	-	-	-	9.31 ± 2.357	-	-	-
11.489	Diazinon	5.84 ± 1.742	4.62 ± 0.079	-	-	6.30 ± 1.296	-	-	-	-
12.935	Metribuzin	-	2.02 ± 0.505	-	1.99 ± 0.724	-	-	-	-	-
13.481	Profenofos	17.00 ± 5.762	14.31 ± 7.891	-	-	1.57 ± 0.567	-	-	-	-
13.689	Metalaxyl	6.59 ± 0.329	4.40 ± 0.513	5.99 ± 1.894	-	11.84 ± 0.848	10.14 ± 0.178	-	-	0.89 ± 0.263
14.681	Triazophos	11.49 ± 1.360	-	4.40 ± 0.602	-	-	-	-	-	-
15.302	Chlorpyrifos-methyl	37.22 ± 1.600	39.16 ± 1.013	34.18 ± 10.156	30.05 ± 0.920	10.70 ± 0.909	21.26 ± 3.665	9.91 ± 1.931	9.83 ± 1.735	-
16.799	Pendimethalin	1.88 ± 0.872	3.53 ± 0.629	-	-	-	-	15.80 ± 2.934	-	-
16.894	Penconazole	-	-	-	29.77 ± 4.301	-	-	-	6.80 ± 1.794	-
18.712	Cypermethrin	1.14 ± 0.011	0.80 ± 0.122	7.68 ± 0.987	1.18 ± 0.199	-	-	-	-	-
20.307	Flusilazole	-	-	-	-	8.12 ± 1.406	1.21 ± 0.238	-	-	-
24.418	Propargite	16.71 ± 2.981	16.11 ± 1.987	-	14.89 ± 0.270	-	-	3.26 ± 1.021	8.09 ± 0.612	-
27.873	Pyriproxyfen	-	-	6.28 ± 0.879	-	57.12 ± 11.246	-	-	-	4.06 ± 0.969
Total		112.29	98.36	62.41	77.88	122.41	41.92	28.97	24.72	4.95

Data presented as mean ± standard deviation

-Not detected

#### Winter season

During winter 2022, pollen (Ismailia) and beeswax (El-Noubariya and Toshka) samples had great amounts and number of pesticide residues (Table 5). Abundant numbers of pesticides were detected in beeswax from El-Noubariya and Toshka compared to Ismailia. More pesticides were detected in honey from El-Noubariya than in the other two locations. The common pesticides in pollen, beeswax, and honey were diazinon, propargite, profenofos, chlorpyrifos-methyl, flusilazole, penconazole, and cypermethrin ranging from 0.06 ± 0.002 to 16.86 ± 0.950 ng/g in the analyzed samples from the three locations.

**Table 5:** Pesticide residues (ng/g) in pollen, beeswax, and honey samples collected from studied areas during winter season of 2022

Rt (min)	Pesticide	El-Noubariya			Ismailia			Toshka		
		Pollen	Beeswax	Honey	Pollen	Beeswax	Honey	Pollen	Beeswax	Honey
6.854	Azinphos-ethyl	-	-	-	0.51 ± 0.101	-	-	-	2.86 ± 0.386	-
10.918	Atrazine	-	-	-	0.18 ± 0.031	-	-	-	-	-
11.489	Diazinon	-	2.06 ± 0.160	1.32 ± 0.115	5.27 ± 0.655	-	0.06 ± 0.002	-	0.19 ± 0.061	-
11.583	Malathion	3.07 ± 0.561	-		-	-	-	-	-	-
12.935	Metribuzin	-	4.69 ± 0.475	-	-	-	-	0.05 ± 0.007	-	-
13.481	Profenofos	-	11.76 ± 0.974	3.13 ± 0.235	-	11.35 ± 0.026	0.27 ± 0.088	1.80 ± 0.483	4.21 ± 0.590	-
15.302	Chlorpyrifos-methyl	3.94 ± 0.056	12.53 ± 1.390	0.97 ± 0.241	5.17 ± 3.125	4.12 ± 0.496	1.96 ± 0.160	1.71 ± 0.018	0.30 ± 0.010	0.47 ± 0.022
15.635	Imazalil	-	-	-	-	7.22 ± 2.212	-	-	0.27 ± 0.009	-
15.904	Iprodione	-	1.87 ± 0.127	-	1.08 ± 0.477	-	-	-	-	-
16.894	Penconazole	7.59 ± 1.736	2.10 ± 0.532	-	0.83 ± 0.133	2.61 ± 1.236	-	0.15 ± 0.012	1.78 ± 0.021	-
16.902	Cyhalothrin	6.44 ± 0.048	4.00 ± 0.190	-	-	-	-	-	-	-
18.712	Cypermethrin	-	2.28 ± 0.171	3.79 ± 0.136	0.57 ± 0.143	4.22 ± 2.080	-	0.13 ± 0.009	0.15 ± 0.001	-
20.307	Flusilazole	4.07 ± 0.363	-	-	-	0.63 ± 0.256	-	-	-	0.38 ± 0.085
20.454	Fludioxonil	-	-	-	0.37 ± 0.079	-	-	-	-	-
20.562	Fenvalerate	3.56 ± 1.349	-	-	-	-	-	-	0.08 ± 0.012	-
24.418	Propargite	11.37 ± 3.222	16.68 ± 0.950	-	1.89 ± 0.773	0.74 ± 0.070	-	0.60 ± 0.016	0.74 ± 0.025	-
Total		40.04	57.97	9.21	15.87	30.89	2.23	4.50	10.58	0.85

Data presented as mean ± standard deviation

-Not detected

**Fig. 2:**
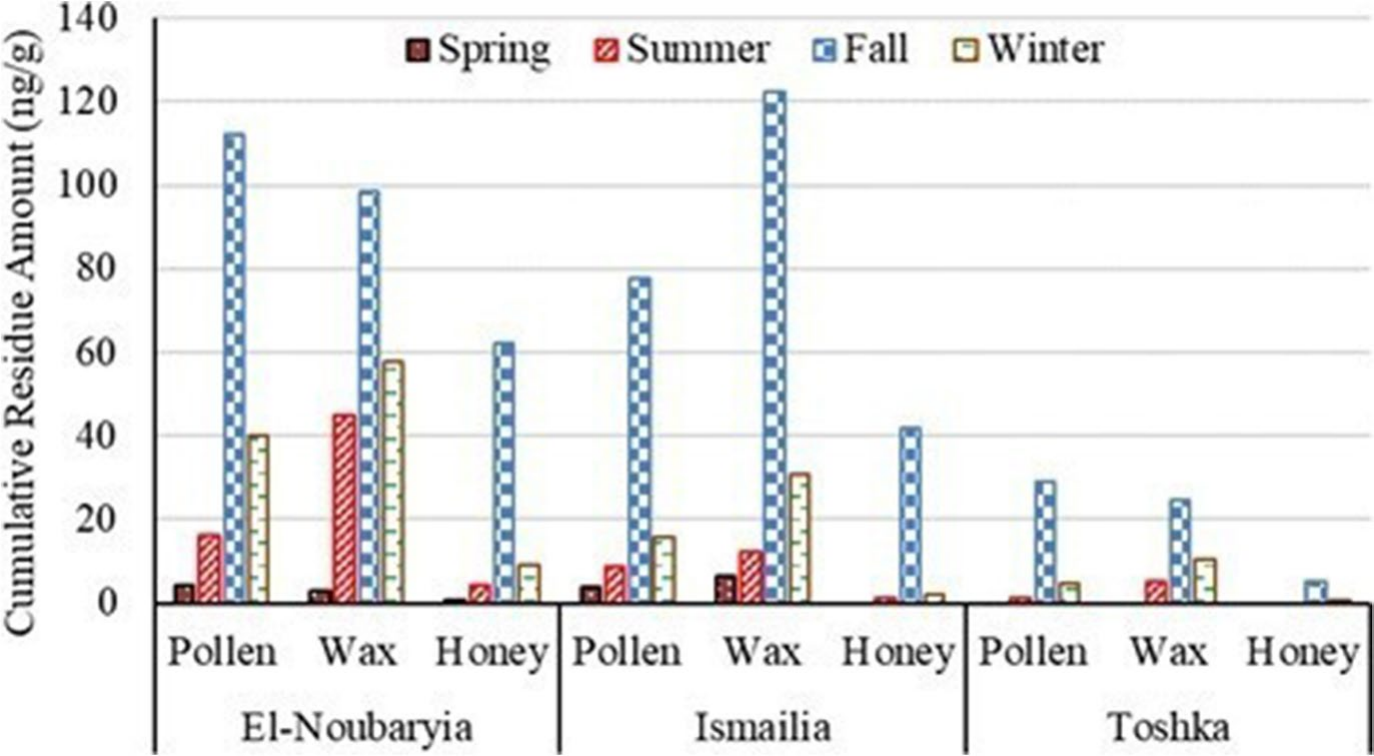
Cumulative residue amounts in ng/g of pesticides in pollen, beeswax, and honey samples collected from El-Noubariya, Ismailia, and Toshka during spring, summer, fall, and winter seasons

Across the four seasons, samples from El-Noubariya had the greatest content of pesticide residues followed by samples from Ismailia, while samples from Toshka had the least content of pesticides (Fig. 2). More pesticides were detected in samples collected during fall and winter than in summer and spring. During the four seasons, beeswax samples had the greatest amounts of residues followed by pollen and then honey samples. Also, the fungicide phenylconazole group was the most frequently detected in all examined samples along with organophosphate insecticides.

### Health quotient (HQ) and index (HI)

Mean daily intake (MDI) of detected residues was estimated considering the average concentrations of the pesticides in honey and the consumed amount of honey by an adult person and his average body weight. The HQ and hazard index (HI) were calculated based on the values of the ARfD of the pesticide residue. Results reported herein showed that each honey sample has more than one residue, and the HQ was estimated for individual ones (Table 6). Then, their values were summed to get the HI. It was clear that HIs for pesticides were less than one that specified no health risk (Choudhury & United States Enmvironment Protecion Agency, 2000).

**Table 6:** Health hazard quotient (HQ) and health index (HI) of pesticide residues in honey samples

Common Name	ArfD* (ng/g)	El-Noubariya			Ismailia			Toshka		
		Conc (ng/g)	MDI	HQ	Conc (ng/g)	MDI	HQ	Conc (ng/g)	MDI	HQ
Spring 2021										
Flusilazole	20	0.61	0.008	0.0004	-	-	-	-	-	
Total HQ (HI)				0.0004						
Summer 2021										
Chlorpyrifos-methyl	30	2.11	0.028	0.0009	0.81	0.0108	0.0003	-	-	-
Fludioxonil	U^#^	0.21	0.0028	-	0.21	0.0028	-	-	-	-
Total HQ (HI)				0.0009			0.0003			
Autumn 2021										
Azinphos-ethyl	75	3.88	0.0517	0.0007	-	-	-	-	-	-
Chlorpyrifos-methyl	30	34.18	0.4557	0.0152	21.26	0.2835	0.0095	-	-	-
Cypermethrin	50	7.68	0.1024	0.0020	-	-	-	-	-	-
Flusilazole	20	-	-	-	1.21	0.0161	0.0008	-	-	-
Metalaxyl	U	5.99	0.0667	-	10.14	0.1352	-	0.89	0.0119	-
Pyrimethanil	U	-	-	-	9.31	0.1241	-	-	-	-
Pyriproxyfen	100	6.28	0.0837	0.0008	-	-	-	4.06	0.0541	0.0005
Triazophos	U	4.40	0.0587	-	-	-	-	-	-	-
Total HQ (HI)				0.0187			0.0103			0.0005
Winter 2022										
Chlorpyrifos-methyl	30	0.97	0.0129	0.0004	1.96	0.0261	0.0009	0.47	0.0063	0.0002
Cypermethrin	50	3.79	0.0505	0.0010	-	-	-	-	-	-
Diazinon	30	1.32	0.0176	0.0006	0.06	0.0008	0.00003	-	-	-
Flusilazole	20	-	-	-	-	-	-	0.38	0.0051	0.0003
Profenofos	1000	3.13	0.0417	0.0004	0.27	0.0036	0.000004	-	-	-
Total HQ (HI)				0.0024			0.000934			0.0005

*References (Australian Pesticides and Veterinary Medicines Authority, 2023; El-Nahhal, 2020)

^#^*U*, the ArfD value is considered to be unnecessary due to its low oral toxicity and the absence of any developmental toxicity

## Discussions

The employed analytical method was suitable for the estimation of pesticide residues in bee products where the CV values ranged from 2.92 to 5.77% and from 4.15 to 11.62% for intra- and inter-assay, respectively. These values were within the pre-defined limits by International Conference on Harmonization (ICH) for the suitability of the analytical method (Branch, 2005) and less than 21% of the reported CV% by Shendy et al. (2016). Also, the recovery % ranged from 60.74 to 90.45% for honey, from 65.54 to 95.12% for pollen, and from 82.00 to 114.55% for beeswax samples, which were in agreement with results by other researchers who reported recoveries from 68 to 126% (Blasco et al., 2004; Calatayud-Vernich et al., 2018; Eissa et al., 2014; Fernandez et al., 2002; Li et al., 2015; Panseri et al., 2014; Perugini et al., 2018; Rissato et al., 2007; Wang et al., 2022).

In current study, samples from two out of the three regions had pesticides, and the third region (Toshka in the south of Egypt) showed less amount and number of pesticides in honey, pollen, and wax. Pollen and beeswax samples had greater numbers and amounts of pesticide residues than honey. Accordingly, beeswax components can maintain fat-soluble acaricides more than honey (Bogdanov, 2006). Also, varroacides that were used in the hives were detected more in beeswax than in pollen unlike the externally derived pesticides (Johnson et al., 2010). Pesticides can be found in pollen and beeswax such as in-hive treatments and agrochemicals (Végh et al., 2023). The analyzed pollen samples contained high numbers of pesticides year-round. Similarly, pollen samples from bee colonies showed residues of insecticides, miticides, fungicides, and herbicides (Krupke et al., 2012).

Beeswax comprises fatty acids, fatty alcohols, and paraffinic hydrocarbons that could preserve pesticide residues (Perugini et al., 2018). The accumulation of pesticide residues in beeswax is well documented than other bee matrices (Al-Naggar et al., 2015; Calatayud-Vernich et al., 2018; Chauzat & Faucon, 2007; Fernandez et al., 2002; Panseri et al., 2014; Perugini et al., 2018; Végh et al., 2023). Therefore, beeswax might pose not only risks for bees but also for humans (Al-Naggar et al., 2015; El-Nahhal, 2020; Irungu et al., 2016; Perugini et al., 2018; Végh et al., 2023). In our study, minor amounts of pesticides were detected which implies that low use or no pesticides were applied inside bee colonies. Also, pollen and beeswax samples had similar pesticides which means that their sources were similar. Pesticides transferred to colonies with contaminated bee bodies or pollen loads, and then accumulated in beeswax.

Beeswax contamination could result from both in-hive acaricide treatment and environmental pollution (Chauzat & Faucon, 2007) with the fact that 40 of the detected pesticides were systemic (Johnson et al., 2010; vanEngelsdorp et al., 2009). Beeswax contained significant levels of miticides (i.e., coumaphos, chlorfenvinphos, fluvalinate, and acrinathrin) that were used in beekeeping (Calatayud-Vernich et al., 2018). Also, pollen samples had greater concentrations of profenofos, chlorpyrifos, malathion, and diazinon insecticides compared to honey samples (Al-Naggar et al., 2015). Pollen was largely polluted with miticides and insecticides that were used in the agricultural practices such as chlorpyrifos and acetamiprid with significant amounts in apiaries located in intensive farming areas (Calatayud-Vernich et al., 2018).

Significantly, few amounts and numbers of pesticides were detected in honey samples compared to pollen and beeswax. Moreover, the detected amounts do not pose any health risks to consumers. Likewise, previous studies showed no harmful effects of pesticide residues in honey (Davodpour et al., 2019; Eissa et al., 2014). The low levels of residues in honey could be a result of the filtration process that is done by bee workers during honey manufacturing from nectar (Wallner, 1999). Additionally, the instability of some pesticides leads to rapid disintegration after application. Indeed, MRLs for pesticide residues in honey are limited to 0.01 mg/kg in the EU for pesticides with no fixed MRL (Bogdanov, 2006; European Commission, 2022). In line with our study, honey samples collected from Qalyoubia, Sharqya, and Ismailia Governorates (Egypt) had residues of HCB, d-HCH, endrin, dieldrin, heptachlor epoxide, c-chlordane, endosulfan, p,p′-DDD, p,p′-DDT, methoxychlor, cyhalothrin, permethrin, and fenvalerate with amounts ranged from 0.06 ± 0.002 to 18.70 ± 1.041 ng/g (Malhat et al., 2015). Also, hazard quotients (HQs) in all examined honey samples all year around were less than one which indicate no risk to humans similar to what was concluded by Al-Naggar et al. (2015).

## Conclusions

This study reported more pesticide residues in samples from El-Noubariya and Ismailia, the two locations in the north of Egypt, than the location in the south of Egypt (Toshka). The environment in the south of Egypt seems to be cleaner with less pesticide usage than those in the north. Pollen and beeswax samples had more pesticides than honey. Also, pesticide residues detected during fall and winter were more than in spring and summer. This suggests the accumulation of pesticides in bee products during late beekeeping seasons (autumn-winter) rather than active seasons (spring-summer). The phenylconazole fungicides and organophosphate insecticides were frequently detected in the examined samples. Also, HQ values in all examined honey samples were less than the level that causes risk to humans. This study confirms the suitability of using bee products to monitor environmental contamination in areas with desert nature.

## Data Availability

Not applicable.
